# Supplementary material for: The Impact of Ag Nanoparticles and CdTe Quantum Dots on Expression and Function of Receptors Involved in Amyloid-β Uptake by BV-2 Microglial Cells
Source: Materials (Basel). 2020 Jul 20;13(14):3227. doi: 10.3390/ma13143227 (PMC7412234; doi:10.3390/ma13143227)
Supplement: Supplementary file 1 [file materials-13-03227-s001.pdf]

materials

Supplementary Materials: The Impact of Ag Nanoparticles and CdTe Quantum Dots on Expression and Function of Receptors Involved in Amyloid-β Uptake by BV-2 Microglial Cells

Katarzyna Sikorska [1,*], Iwona Grądzka [1], Iwona Wasyk [1], Kamil Brzóska [1], Tomasz M. Stępkowski [1,†], Malwina Czerwińska [1] and Marcin K. Kruszewski [1,2]

[1] Centre for Radiobiology and Biological Dosimetry, Institute of Nuclear Chemistry and Technology, Dorodna 16, 03-195 Warsaw, Poland; i.gradzka@ichtj.waw.pl (I.G.); iwonawasyk@gmail.com (I.W.); k.brzoska@ichtj.waw.pl (K.B.); t.stepkowski@cent.uw.edu.pl (T.M.S.); m.wasilewska@ichtj.waw.pl (M.C.); m.kruszewski@ichtj.waw.pl (M.K.K.)

[2] Department of Molecular Biology and Translational Research, Institute of Rural Health, Jaczewskiego 2, 20-090 Lublin, Poland

* Correspondence: k.sikorska@ichtj.waw.pl; Tel.: +48-22-504-12-38

† Present address: Centre of new technologies, S. Banacha 2c, 02-097 Warsaw, Poland.

Table 1. The *Msr1*, *Cd36*, *Ager* and *Cd33* gene expression of BV-2 cells after treatment with NPs for 6 h (real-time PCR).

Sample	*Gene*	Mean Rq value	Rq Min	Rq Max	*P* Value
**control**	*Ager*	1	0.757	1.322	1
	Cd33	1	0.735	1.361	1
	Cd36	1	0.573	1.744	1
	Msr1	1	0.487	2.055	1
**AgNPs 5 μg mL^{-1}**	*Ager*	1.107	0.699	1.753	0.764
	Cd33	0.960	0.736	1.252	0.870
	Cd36	0.860	0.424	1.746	0.787
	Msr1	1.014	0.532	1.932	0.982
**AgNPs 50 μg mL^{-1}**	*Ager*	0.959	0.818	1.125	0.836
	Cd33	0.855	0.653	1.120	0.545
	Cd36	0.973	0.592	1.599	0.952
	Msr1	1.233	0.668	2.278	0.721
**CdTeQDs 0.1 μg mL^{-1}**	*Ager*	0.857	0.706	1.041	0.482
	Cd33	0.955	0.751	1.214	0.849
	Cd36	1.280	0.703	2.331	0.629
	Msr1	0.957	0.478	1.916	0.943
**CdTeQDs 10 μg mL^{-1}**	*Ager*	0.600	0.351	1.024	0.238
	Cd33	0.836	0.625	1.117	0.504
	Cd36	1.462	0.919	2.324	0.417
	Msr1	0.944	0.558	1.598	0.917

Data were compared to untreated control. Results are mean of three independent experiments, RqMin and RqMax are also shown. Rq (relative quantification) is a relative change in a gene expression, see Materials and Methods section.

© 2020 by the authors. Submitted for possible open access publication under the terms and conditions of the Creative Commons Attribution (CC BY) license (http://creativecommons.org/licenses/by/4.0/).
